# Urinary MicroRNA Profiling in the Nephropathy of Type 1 Diabetes

**DOI:** 10.1371/journal.pone.0054662

**Published:** 2013-01-24

**Authors:** Christos Argyropoulos, Kai Wang, Sara McClarty, David Huang, Jose Bernardo, Demetrius Ellis, Trevor Orchard, David Galas, John Johnson

**Affiliations:** 1 Renal and Electrolyte Division, Department of Medicine, University of Pittsburgh, Pittsburgh, Pennsylvania, United States of America; 2 Institute for Systems Biology, Seattle, Washington, United States of America; 3 Luxembourg Center for Systems Biomedicine, University of Luxembourg, Esch-sur-Alzette, Luxembourg; 4 Department of Epidemiology, Graduate School of Public Health, University of Pittsburgh, Pittsburgh, Pennsylvania, United States of America; 5 Children’s Hospital of Pittsburgh, Pittsburgh, Pennsylvania, United States of America; 6 Pacific Nothwest Diabetes Research Institute, Seattle, Washington, United States of America; Baker IDI Heart and Diabetes Institute, Australia

## Abstract

**Background:**

Patients with Type 1 Diabetes (T1D) are particularly vulnerable to development of Diabetic nephropathy (DN) leading to End Stage Renal Disease. Hence a better understanding of the factors affecting kidney disease progression in T1D is urgently needed. In recent years microRNAs have emerged as important post-transcriptional regulators of gene expression in many different health conditions. We hypothesized that urinary microRNA profile of patients will differ in the different stages of diabetic renal disease.

**Methods and Findings:**

We studied urine microRNA profiles with qPCR in 40 T1D with >20 year follow up 10 who never developed renal disease (N) matched against 10 patients who went on to develop overt nephropathy (DN), 10 patients with intermittent microalbuminuria (IMA) matched against 10 patients with persistent (PMA) microalbuminuria. A Bayesian procedure was used to normalize and convert raw signals to expression ratios. We applied formal statistical techniques to translate fold changes to profiles of microRNA targets which were then used to make inferences about biological pathways in the Gene Ontology and REACTOME structured vocabularies. A total of 27 microRNAs were found to be present at significantly different levels in different stages of untreated nephropathy. These microRNAs mapped to overlapping pathways pertaining to growth factor signaling and renal fibrosis known to be targeted in diabetic kidney disease.

**Conclusions:**

Urinary microRNA profiles differ across the different stages of diabetic nephropathy. Previous work using experimental, clinical chemistry or biopsy samples has demonstrated differential expression of many of these microRNAs in a variety of chronic renal conditions and diabetes. Combining expression ratios of microRNAs with formal inferences about their predicted mRNA targets and associated biological pathways may yield useful markers for early diagnosis and risk stratification of DN in T1D by inferring the alteration of renal molecular processes.

## Introduction

Diabetic nephropathy (DN) is the leading cause of End Stage Renal Disease (ESRD) in the Western world, accounting for more than 40% of cases. Patients with either type 1 (T1D) or 2 (T2D) diabetes are at risk of DN, but the disease burden is higher in the former group [1]. Hence a better understanding of the factors affecting disease progression(2) from hyperfiltration to microalbuminuria(MA), dipstick positive macroalbuminuria, impaired filtration and ESRD in patients with T1D is urgently needed. The molecular pathophysiology [2] of diabetic nephropathy is multifactorial, involving hemodynamic factors (Vascular Endothelial Growth Factor, renin-angiotensin-aldosterone and endothelin systems), proinflammatory (e.g. Interleykin IL-1, 6,18) and profibrotic cytokines (such as Transforming Growth Factor beta, TGFβ) as well as other biochemical derangements (polyol, Protein Kinase C). Nevertheless, the manner in which these diverse molecular processes are regulated, resulting in distinct clinical courses of individual patients remains poorly defined.

In recent years microRNAs (miRNAs), a family of short (average of 22nt long), naturally occurring, small antisense non-coding RNAs have emerged as important post-transcriptional regulators of gene expression (see review [3]). First described in *C. elegans* [4], they have since been discovered to be widely distributed, endogenous controllers of gene and protein expression by binding to the 3′-untranslated region of specific mRNAs and interfering with protein synthesis by inducing mRNA degradation or repressing translation [5], [6]. A number of these miRNAs have also been identified in the extracellular environment. As they may regulate a significant portion of the transcriptome and proteome, considerable attention has focused on miRNAs as mediators or biomarkers of illness. Previous work in diabetic renal disease (see reviews [7]–[10]) performed in cell cultures, animal models or formalin fixed human biopsy material has linked a number of miRNAs to the development of nephropathy. Nevertheless, there have been no comprehensive studies examining miRNA signatures in human urine in relation to either longitudinal clinical outcomes and/or the level of urinary albumin, which is the current gold standard for detecting and staging diabetic nephropathy in the clinic. The goal of this pilot study is to identify the differences on urinary miRNA profiles in patients with long standing T1D who were either free from diabetic nephropathy or had developed variable degrees of albuminuria after long follow-up. In addition, by integrating experimentally verified alterations on urinary miRNAs with miRNA target prediction databases, we translated the changes of miRNAs into hypotheses about signalling pathways associated with nephropathy induced by diabetes.

## Materials and Methods

### Patients and Samples

Urine samples from participants in the Pittsburgh Epidemiology of Diabetes Complications (EDC) study were examined. The EDC study is a historical prospective cohort which recruited patients from Children’s University Hospital of Pittsburgh Registry of all cases of T1D, diagnosed or seen within a year of diagnosis between January 1^st^ 1950 and May 31^st^ 1980. Participants were followed thereafter with repeat exams biennially for 10 years and again at 18 years. Follow up of all participants in the EDC was censored for this analysis on December 31^st^ 2000.

In the EDC, diabetic renal disease was characterized in terms of its progression from a normoalbuminuric urine examination to progressively higher amounts of albumin in the urine (microalbuminuria) to overt nephropathy. Microalbuminuria was defined as 20–200 µg/min in at least two of three timed urines (24hr, overnight, and 4 hr clinic visit) and was further classified as *intermittent*(IMA) or *persistent* (PMA) on the basis of subsequently reverting to normoalbuminuria or persisting at least to microalbuminuria level throughout further follow up respectively. *Diabetic nephropathy* was defined as an albumin excretion rate >200 µg/min in at least two of three timed urine collections (24-h, overnight, and post-clinic). In the absence of urine, a serum creatinine >2 mg/dl or renal failure was accepted as an alternative diagnostic criterion for overt nephropathy. For the purpose of this report we analyzed urine from matched samples of a) diabetic patients who never developed microalbuminuria or nephropathy after prolonged (25 year) follow – up vs. those with DN and b) patients who developed IMA matched against EDC participants who developed PMA. In the case of the N v DN group we collected a single urine sample, while two samples were analyzed from patients who developed microalbuminuria : a (baseline)urine sample from the last visit which tested negative for albumin and the subsequent (follow-up, albuminuric) sample which was collected 2 years after the first. Matching in the 2 sample sets was independently carried out on the basis of age, sex, duration of disease and levels of Hemoglobin A1c (HBA1c) to account for unmeasured confounders.

### RNA Isolation

The RNA from urine was isolated using the miRNeasy kit (Qiagen, Germantown, MD). In brief, 700 µl of QIAzol reagent was added to 200 µl of urine sample. The sample was mixed in a tube followed by adding 140 µl of chloroform. After mixing vigorously for 15 seconds, the sample was then centrifuged at 12,000×g for 15 minutes at 4°C. The upper aqueous phase was carefully transferred to a new collection tube, and 1.5 volume of ethanol containing binding buffer from the kit was added and mixed. The sample was then applied directly to a silica membrane containing column and the RNA was retained and cleaned by using buffers provided in the kit. The immobilized cleaned RNA was then eluted from the membrane into a collection tube with a low salt elution buffer or water. The quality and quantity of the RNA was evaluated by 260/280 ratio and Agilent 2100 Bioanalyzer (Agilent Technologies, Santa Clara CA).

### miRNA Profiling

In brief, the cDNA was generated from 20 µl of RNA using buffer and enzyme provide in the Qiagen kit. After incubating the cDNA synthesis reaction at 42°C for 60 minutes, the cDNA was diluted to 8 ml with SYBR containing PCR reagents from Exiqon and water. The plates were then loaded onto ABI 7900HT real-time PCR system and the threshold cycle (C_t_) was measured with standard methods. Exiqon miRNA qPCR panels 1 and 2 (Version 1) were used, that included probes for 748 unique miRNA. Each miRNA species was assayed once per panel with the exception of miR-423-5p, miR-103, miR-191 and the three non-coding RNA species U6, SNORD38B and SNORD39A for which duplicate reactions was set up as per panel manufacturer instructions. Although suggested as reference genes (biological controls) by the panel manufacturer the 6 microRNAs/small nuclear RNAs were not used as referents during normalization. Nevertheless their presence in multiple technical replicates in any given panel, allowed us to derive panel specific normalization factors which were applied to the raw expression levels of all microRNAs. A single inter-plate calibrator spiked in control (UniSP3) was run 6 times per plate and was used to normalize the expression levels of all miRNAs included in each of the qPCR panels. A second spiked in control (UniSP6) was included in some but not all urine reactions as a dual positive – negative control and was thus not considered in subsequent analyses (including normalization). We also included a no-template negative control in all assays (nine replicates per assay) as per manufacture guidelines. To resolve discrepancies in the nomenclature of miRNA species, we mapped names of miRNAs present in the Exiqon plates to the most current ones in miRBase (version 18, November 2011) and the associated MIMAT accession numbers (Table S1).

### Statistical Analyses

#### Quantification cycle (Threshold Crossing) C_q_ visualization, signal analysis and normalization

In order to classify individual patient samples and visualize the resemblance in the corresponding profiles we applied Principal Component Analysis (PCA) to the corrected C_q_ values obtained from the raw C_q_ measurements after subtracting the quantification cycle number of the spiked-in control. To handle missing data in the expression of miRNAs across samples we applied a specific variety of PCA, i.e. Probabilistic PCA (PPCA) [11] that combines the Expectation Maximization (EM) with PCA to simultaneous estimate missing expression values and the principal components in the dataset. Results of PPCA were plotted as bivariate scatterplots, in which each principal component is plotted against all others. PPCA calculations were performed with the “pcaMethods” package [12] in *bioconductor* [13].

To analyze the difference in miRNA expression within patient groups, we quantified the relative expression level of each miRNA, its normalized threshold cycle difference (ΔC_q_) i.e. the difference between the quantification cycle in the experimental (E) and the reference (R) state : ΔC_q_ = C_q_(E) – C_q_(R), with positive ΔC_q_ values indicating lower concentrations. To ensure a sufficient amount of data for downstream analyses, only those miRNAs that were detected in at least 2/3 of patient samples in each comparison were analyzed.

**Figure 1:**
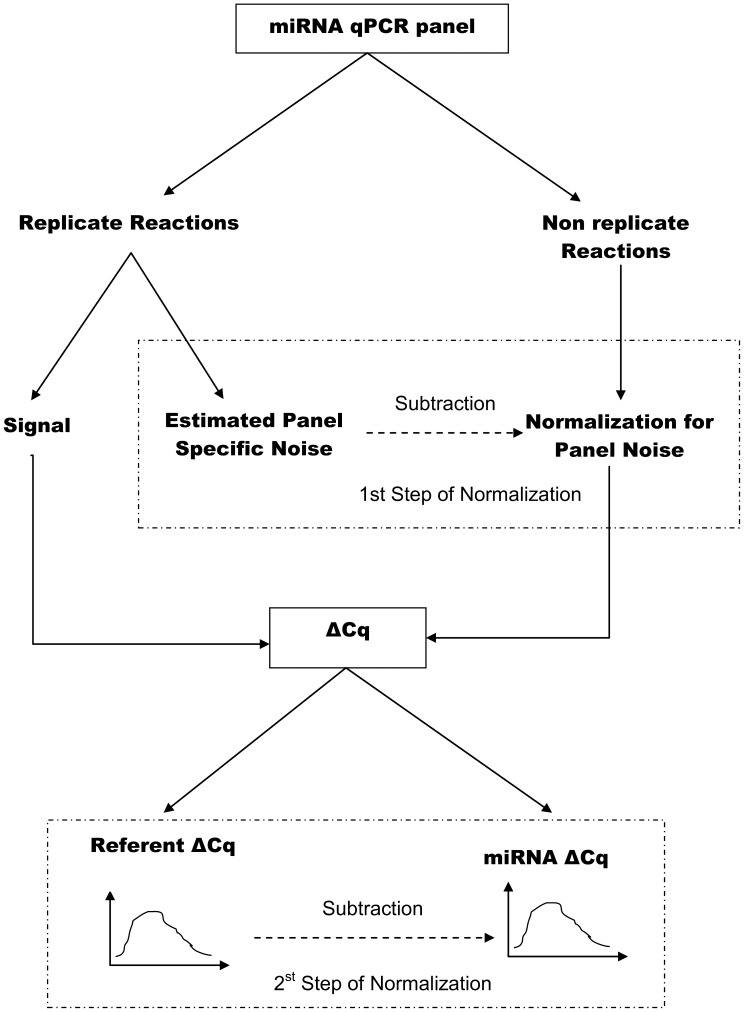
Schematic representation of the normalization procedure and estimation of relative fold changes adopted in the manuscript. Replicate qPCR reactions were analyzed with a hierarchical linear mixed model in order to estimate panel specific correction factors that were subtracted from the raw C_q_ signals of unreplicated reactions (first step), while simultaneously estimating the difference (ΔC_q_) between an experimental and referent state. In the second step, the ΔC_q_ of the spiked in control was subtracted from the non-control ΔC_q_ values to calibrate the relative fold changes according to the Delta-Delta method. Both steps of the normalization procedure acknowledged the uncertainty implicit in estimating the ΔC_q_ of both control and non-control signals (shown as a density plot at the bottom part of the figure), by performing this subtraction probabilistically i.e. by Monte Carlo methods.

A mixed effects model was used simultaneously accounting for matching patients within pairs while normalizing ΔC_q_ values for PCR related factors. Normalization of quantification cycle signals occurred in two steps (**Figure 1** **)**: first, we developed a regression model that utilized the multiple replicates in the qPCR panels to decompose the corresponding measurements into signal and (panel/PCR) specific noise factors. Secondly, the difference in the expression level of the spiked in (UniSp3) control was used to calibrate *relative fold changes* (*FC*) by the Delta-Delta method [14], [15] as: FC  = 2^−ΔΔCq^, where ΔΔC_q_ = ΔC_q_(miRNA) – ΔC_q_(UniSp3). The parameters of the regression model were estimated from a Bayesian probabilistic viewpoint, a decision justified by the exploratory, hypothesis generating [16] nature of this work and the amenability of the complex mixed models utilized to Bayesian computational methods. In this study we used “objective”, likelihood-dominated, non-informative priors [17] due to the lack of previous information that could be used to specify prior beliefs for the levels of the miRNAs examined.

Bayesian models were programmed in the BUGS language (Text S1 sections Bayesian Computations and WinBUGS code) [18]. Statistics (means, standard errors) of posterior probability MCMC samples were used to summarize inferences about individual ΔC_q_ values, while the degree to which each estimated ΔC_q_ differs from zero was quantitated by means of *symmetric pseudocontour* probabilities which can be viewed as Bayesian analogs of p-values [19]. 95% Posterior Density Credible (symmetric) Intervals (CrI) were used to provide a range of values in which the estimated ΔC_q_ lie with a probability of 95%.

#### miRNA target functional profiling

To infer putative targets of differentially expressed miRNAs we utilized three different algorithms: miRanda (release August 2010) [20], TargetSCan (release 6, November 2011) [21] and miRDB (version 4.0, January 2012) [22]. In order to declare a specific mRNA as a target of a given miRNA species, we required that at least 2 of the 3 databases predict the latter to bind to the former. To leverage the quantitative urinary expression profiles and miRNA target database information into more concrete predictions we appealed to a biochemical argument based on Hill plots. In this approximation for the interaction between miRNA and mRNA, the fraction of the bound sites (*θ*) is related to the free ligand concentration (*L*) and the dissociation constant (*K_d_*) by the *logistic equation*:

A change in the ligand concentration between an experimental state (*L_E_*) and the reference (*L_R_*) is related to a change in the fraction of bound sites which can be expressed in terms of the relative fold change . Hence, by the above expression:

To the extent that miRNAs function as negative regulators of mRNA translation a positive log-odds ratio (larger bound fraction) would imply a propensity for the target mRNA expression to be reduced in the experimental state.

To synthesize the evidence from multiple *ΔΔC_t_* values of miRNAs targeting a specific gene we used the means and standard errors from the MCMC simulations as input to *random effects meta-analyses*. Such techniques, allow one to test the hypotheses that a given sample of *ΔΔC_q_*’s (“treatment effects”) follows a distribution with a mean that departs from zero. In such a case, one would expect the mRNA profile to deviate to a direction opposite to the miRNA distributional mean (under-expressed miRNA implies overexpression of the cognate mRNA and vice versa). Hence, by considering the log-odds ratios for all putative miRNA-mRNA pairs we estimated functional expression profiles for the miRNA targets for post hoc exploration (enriched term analysis) in the REACTOME [23] and Gene Ontology Project [24].

## Results

### Patients and Measurements

Baseline characteristics of patients included in this study are shown in Table 1. In total we studied 40 patients with T1D: 10 who never developed diabetic renal disease (N) matched against 10 patients who went on to develop overt nephropathy (DN), 10 patients with intermittent microalbuminuria (IMA) matched against 10 patients with persistent (PMA) microalbuminuria. In general, patients were well matched within each of the two comparison groups (IMA vs. PMA, N vs. DN) in terms of their demographics and glycemic control (HBA1c). Roughly 50% of patients with DN and an equivalent proportion of patients without renal disease had at least one diabetic complication (most commonly peripheral neuropathy). On the other hand, patients at the microalbuminuria group were free of diabetic complications at the time of urine collection. The majority of the patients in this cohort were not on inhibitors of the angiotensin system (i.e. Angiotensin Enzyme Inhibitors or Angiotensin Receptor Blockers), with the exception of patients with overt nephropathy who were receiving them (8/10 patients). Furthermore, these patients were more likely to receive additional agents for blood pressure control, paralleling the severity of their renal disease.

**Table 1:** Patient Demographics.

	Group A		Group B	
	Clinical Classification		Clinical Classification	
	Normal	Overt Nephropathy	Intermittent Microalbuminuria	Persistent Microalbuminuria
**N of subjects**	10	10	10	10
**Samples (collected)**	10	10	20	20
**Samples(profiled)**	10	10	19	14
**Age (yrs)**	42.8±5.1	41.4±6	29.4±6.3	27.5±5.3
**Women**	5	5	5	5
**Duration of Diabetes (yrs)**	34.1±5.8	34.4±6.4	20.7±5.4	21.3±5.8
**CAD (including MI)**	3	1	0	1
**Stroke**	0	0	0	0
**PVD**	2	2	0	1
**Peripheral Neuropathy**	5	5	0	0
**Proliferative Retinopathy**	4	1	1	5
**Hypertension**	1	6	0	0
**HgBA1c (%)**	8.2±1.1	8.2±1.0	9.9±1.9	10.2±2.4
**LDL-c (mg/dl)**	103.5±20.2	106.3±44.2	100.3±20.6	115.9±57.5
**ACEi or ARB therapy**	1	8	0	0
**LDL-c lowering therapy**	1	5	0	0

*Abbreviations:* CAD (Coronary Artery Disease), MI (Myocardial Infarction), PVD (peripheral vascular disease), HgbA1c (Glycosylated Hemoglobin A1c), LDL-c (Low Density Lipoprotein cholesterol), ACEi (Angiotensin Converting Enzyme Inhibitor), ARB (Angiotensin II Receptor Blocker).

Due to the insufficient amount of RNA, we did not obtain good quality mRNA measurement in 6 samples from 3 patient pairs in the PMA sub-group and one urine sample from the IMA group. Nevertheless, reproducibility of un-normalized C_q_ signals from urine in the rest of the samples was high (Dataset S1 for un-normalized C_q_ values). The quality of miRNA measurements was assessed by using the detection probability of controls (Fig. S1) and the reproducibility of un-normalized signals in replicates present in the qPCR panels (Fig. S2). The detection probability of negative (BLANK) and positive (UniSp6 and UniSp3) controls matched the expected ratios (0%, 50% and 100% respectively). The corresponding C_q_ values for the spiked controls were much less variable than those of endogenous (miRNAs and small RNA) controls suggested by the platform manufacturer without evidence of substantial inter-plate variability. Hence fold changes were computed relative to the UniSp3 RNA for all subsequent comparisons.

## Principal Component Analysis

**Figure 2:**
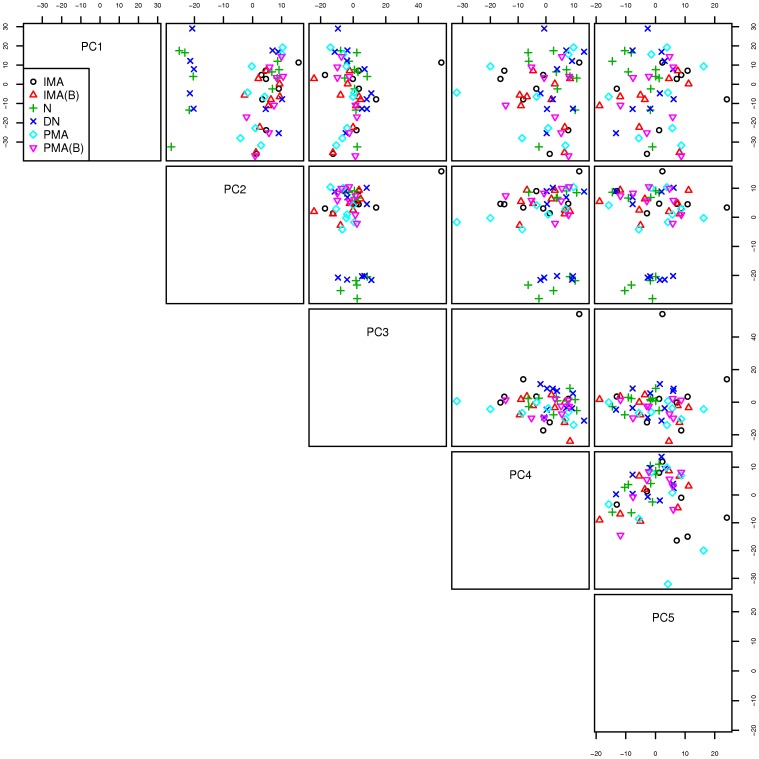
Results of Principal Component Analysis applied to all urine samples analyzed in this study. To present the results of the five dimensional PCA, we utilized bivariate projections in which each component is plotted against all e.g. the second plot in the first row plots the first principal component (PC1) against the second (PC2). Each individual urine sample is color and symbol coded according to the disease classification at the time it was collected. N: patients without nephropathy, DN: patients with overt nephropathy, IMA(B): normoalbuminuric samples from patients who had intermittent microalbuminuria, PMA(B): last normoalbuminuric samples from patients who had persistent albuminuria, IMA: micro-albuminuric samples from patients who had intermittent micro-albuminuria, PMA: micro-albuminuric samples from patients who had persistent microalbuminuria.

A global view of the changes in urinary miRNA profiles according to the clinical classification was performed with PCA and the results for the first five principal components (PC) are shown in **Figure 2**. Samples from Group A (DN vs. N) were grouped together in some projections (e.g. see second plot in the first row depicting PC1 v.s. PC2 ), while in other projections (PC3 vs. PC4 show in the last plot from the left, second row) samples from both comparisons grouped together. Although patients with DN appear to form a cluster distinct from those who never developed nephropathy (projections PC2 v.s.PC3-5, in the second row) there was no obvious clustering structure in the profiles of patients with MA at either the baseline or the microalbuminuric state).

**Figure 3:**
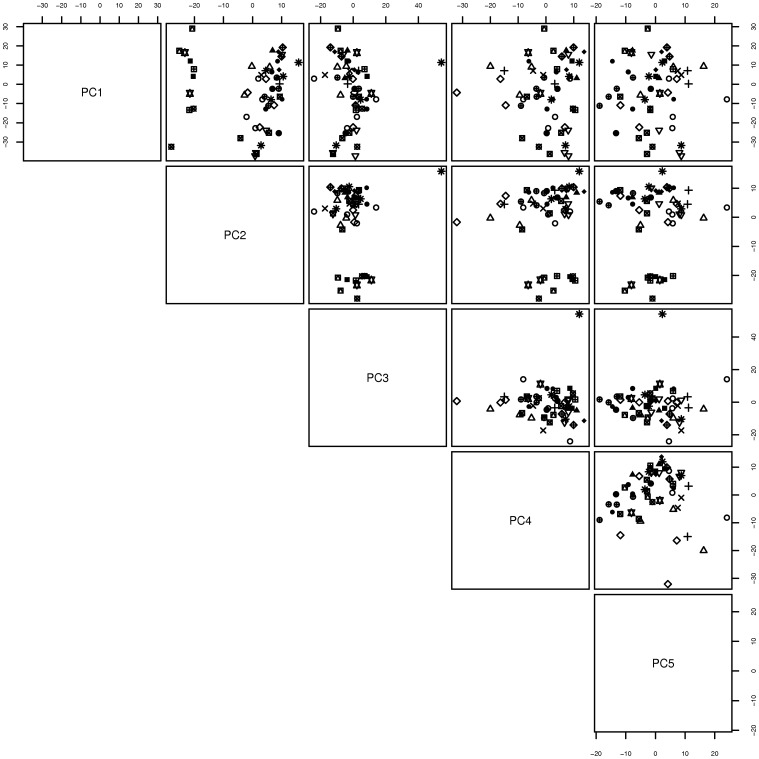
Results of Principal Component Analysis rendered according to pair identification number. This figure utilizes the same bivariate projection setup as Figure 2, but points are symbol coded according to the unique identifier used when matching patients into pairs. For patients with MA who contributed two samples (one at the baseline and one at the microalbuminuric state) there are more than 2 points with the same symbol.

To explore whether patients who had been matched into pairs had similar microRNA profiles we plotted the results of the PCA according to the pair identifier. These results which are shown in **Figure 3** show that patients within pairs have similar profiles (the distance between points with the same plotting symbol appears smaller than the distance of points from patients from different pairs). Taken together these data suggest that there global changes in the miRNA profile associated with the different stages of diabetic nephropathy. Furthermore, the clustering of profiles with patient pairs suggests that there are other determinants of microRNA expression which correlate with the variables we used to match patients during sample selection.

### Comparisons between IMA and PMA

219 miRNAs yielded measurable signals in >75% of the 33 urine samples profiled for this comparison. We observed only a few differences in the baseline samples. Relative to the IMA group, patients with PMA demonstrate decreased *miR-323b-5p* levels (Fold Change (FC) 0.13, 95% CrI: 0.03–0.67, p = 0.014) and increased levels of: *miR-122-5p* (FC: 7.45, 95% CrI: 1.77–32.05, p = 0.006), *miR-429* (FC: 4.72, 95% CrI: 1.14–19.86,p = 0.034), at baseline.

Table 2 shows the miRNAs with altered levels in the MA *samples* relative to baseline. Appearance of micro-albuminuria is associated with decreased levels of *miR-323b-5p* and increased urine concentration of *miR-429.* In Table 3 we summarize the *incremental* FCs of specific miRNA levels in microalbuminuric samples between PMA and IMA patients. Of note, two miRNAs in the PMA patients (miR-373-5p and miR-323b-bp) exhibit concentration changes that are in the opposite direction relative to the changes observed when both IMA/PMA patients manifest MA. There were no further changes observed in the levels of the remaining miRNAs found to be different in Table 2, while only a small number of miRNAs appear to show incremental concentration changes in the microalbuminuric urine from PMA patients. Of note miR-324-3p, a demonstrated a trend towards a higher incremental change in expression level (FC 2.90, 95% CrI: 0.53–17.62,p = 0.11) in patients with PMA.

**Table 2:** Differentially expressed miRNAs between albuminuric and non-albuminuric (reference) samples from patients with MA.

miRNA	Fold Change	95% Credible Interval	P
	Under-expressed		
hsa-miR-323b-5p*hsa-miR-453*	0.07	0.01–0.42	0.0030
hsa-miR-221-3p*hsa-miR-221*	0.15	0.03–0.80	0.0280
hsa-miR-524-5p	0.19	0.04–0.88	0.0350
hsa-miR-188-3p	0.28	0.08–0.98	0.0454

	Over-expressed		
hsa-miR-214-3p*hsa-miR-214*	8.71	1.97–38.05	0.0050
hsa-miR-92b-5phsa-miR-92b*	8.65	1.11–67.46	0.0394
hsa-miR-765	7.22	1.78–30.98	0.0046
hsa-miR-429	5.92	1.42–23.94	0.0136
hsa-miR-373-5phsa-miR-373*	4.50	1.19–17.27	0.0296
hsa-miR-1913	4.37	1.30–15.47	0.0156
hsa-miR-638	3.71	1.02–13.81	0.0464

For miRNAs whose name changed after the introduction of the 18^th^ version of MiRBase, we provide both the previous (in *italics*) and the recent (regular font) name.

**Table 3:** Incremental differential expression of miRNAs between albuminuric samples from patients with persistent microalbuminuria (PMA) relative to patients with intermittent microalbuminuria (IMA).

miRNA	Fold Change	95% Credible Interval	P
	Under-expressed		
hsa-miR-589-5p*hsa-miR-589*	0.05	0.00–0.98	0.048
hsa-miR-373-5phsa-miR-373*	0.07	0.01–0.45	0.007
hsa-mir-520h	0.12	0.02–0.80	0.026
hsa-miR-92a-3p*hsa-miR-92a*	0.14	0.02–0.98	0.048

	Over-expressed		
hsa-miR-323b-5p*hsa-miR-453*	31.51	2.91–368.48	0.0044
hsa-miR-433	16.24	1.39–196.0	0.028
hsa-miR-17-5p*hsa-miR-17*	14.82	1.09–214.0	0.044
hsa-miR-222-3p*hsa-miR-222*	11.22	1.13–102.0	0.036
hsa-let-628-5p	7.59	1.07–52.2	0.044

For miRNAs whose name changed after the introduction of the 18th version of MiRBase, we provide both the previous (in italics) and the recent (regular font) name.

### Comparisons between Patients with and without DN

283 miRNAs yielded measurable signals in >75% of the 20 urine samples from these 10 patient pairs. In Table 4 we summarize the miRNAs with altered expression in the urine of patients with nephropathy. With the exception of miRNA-221-3p, which decreased similar to the comparison of follow up and baseline MA samples, the remaining miRNAs did not demonstrate altered expression in any of the previous comparisons. Finally, there was a trend for miR-589 and miR-323b-5p to be increased in the urine of patients with overt nephropathy. The corresponding FCs were 2.99, (95% CrI: 0.81–9.95, p = 0.087) and 4.45, (95% CrI: 090–29.1, p = 0.08).

**Table 4:** Differentially expressed miRNA between patients who developed overt diabetic nephropathy relative to patients who did not.

miRNA	Fold Change	95% Credible Interval	P
	Under-expressed		
hsa-miR-221-3p*hsa-miR-221*	0.25	0.07–0.86	0.0330

	Over-expressed		
hsa-miR-619	6.98	1.86–27.80	0.0030
hsa-miR-486-3p	6.43	1.36–26.66	0.0290
hsa-miR-335-5p*hsa-miR-335*	5.81	1.70–20.71	0.0050
hsa-miR-552	5.47	1.19–27.50	0.0310
hsa-miR-1912	4.72	1.01–23.92	0.0490
hsa-miR-1224-3p	4.45	1.10–17.48	0.0430
hsa-miR-424-5p*hsa-miR-424*	4.38	1.35–15.13	0.0130
hsa-miR-141-3phsa-miR-141*	3.81	1.29–11.17	0.0140
hsa-miR-29b-1-5phsa-miR-29b-1*	3.03	1.09–8.61	0.0370

For miRNAs whose name changed after the introduction of the 18th version of MiRBase, we provide both the previous (in italics) and the recent (regular font) name.

### miRNA Target Functional Profiling

**Figure 4:**
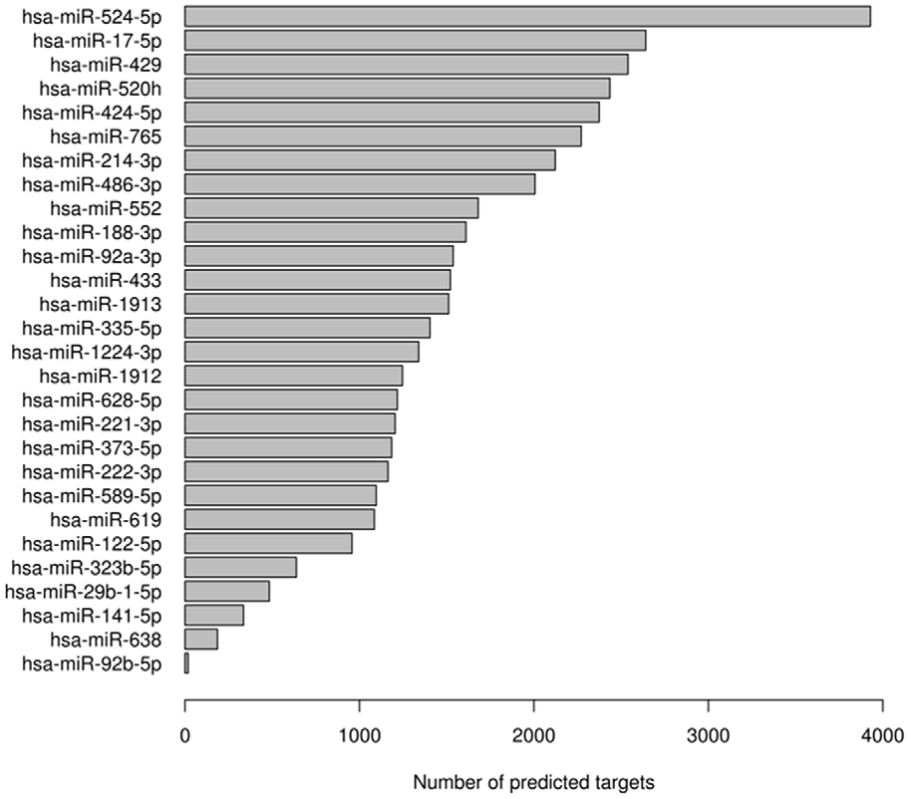
Distribution of the number of mRNAs targeted by differentially regulated microRNAs in diabetic urine.

**Figure 4** summarizes the number of predicted mRNA targets of the differentially expressed miRNAs in diabetic urine based on the prediction databases. Analysis of enriched terms in REACTOME (Table 5) suggest that the predicted miRNA targets map to a distinct pathways involving growth factor signaling, apoptosis, immunity, substrate metabolism, transmembrane transport and certain non-kidney related terms. Furthermore, the identified pathways overlapped considerably between the comparisons of patients with overt nephropathy and normals, and follow-up v.s. baseline samples from MA patients. In the comparisons *within* baseline and follow-up MA samples we found only a few (<80) targets mapping to annotated REACTOME pathways, thus precluding a meaningful assessment with this structured vocabulary. The results from Gene Ontology (GO) analysis (Datasets S2, S3, S4, S5) were consistent with REACTOME, and also identified enrichment of terms relating to nitrogen compound metabolism, Golgi/membrane/ER vesicle recycling, ubiquitin-dependent degradation, cell adhesion and cell adhesion. In addition, GO analyses also suggested the enrichment of renal (GOBPID: 0072166, p = 0.03) and non-renal developmental (GOBPID:0048557, p = 0.0002, GOBPID:0060174, p = 0.002) pathways, myoblast determination (GOBPID:007518, p = 0.0002) innate immunity (GOBPID:0002717, p = 0.001) and free radical generation/oxidative stress (GOBPID:0071371/0071450-51, p = 0.01).

**Table 5:** REACTOME pathway terms enriched in targets of differentially expressed miRNAs.

	Albuminuric vs Normoalbuminuric in the MA group		Overt vs Normal	
Pathway	P-value	Fraction	P-value	Fraction
*Signal Transduction*				
Signaling by SCF-KIT	0.006	18/76	0.001	41/76
Signaling by Insulin receptor	0.009	23/109	<0.001	65/109
Signaling by NGF	0.016	38/212	<0.001	119/212
Signaling by Rho GTPases	0.024	24/125	<0.001	71/125
Signaling by ERBB4	0.027	16/76	<0.001	45/76
Signaling by ERBB2	0.035	19/97	<0.001	59/97
Signaling by PDGF	0.040	22/118	<0.001	67/118
Signaling by VEGF	0.041	4/11		
Signaling by EGFR	0.044	20/106	<0.001	64/106
Dowstream signaling of activated FGFR	0.038	19/98	<0.001	61/98
Signaling by BMP			0.001	16/23
Signaling by TGFβ			0.004	11/15
DAG and IP3 signaling			0.010	20/31
PIP3 activates AKT signaling			0.020	15/26
RAF/MAP kinase cascade			0.031	7/10
Signaling by Notch			0.036	13/23
Interaction of integrin α5β3 with fibrillin	0.044	2/3		
Interaction of integrin α5β3 with von Willbrand factor	0.044	2/3		
Integrin cell surface interactions			0.024	40/85
*Cell-Cell Communication*			0.009	57/122
*Cell Cycle*				
G0 and early G1			0.040	12/21
*Metabolism*				
Metabolism of lipids and lipoproteins	0.022	51/305	0.005	132/205
Cysteine formation from homocysteine	0.016	2/2		
Integration of energy metabolism			0.009	45/93
*Metabolism of proteins*				
Post-translational protein modification	0.045	30/173	0.019	76/173
*Transmembrane transport of small molecules*	0.007	67/396	<0.001	189/396
*Membrane trafficking*			0.032	40/84
*Apoptosis*				
Caspase-8 is formed from procaspase-8	0.019	4/9		
*Gene Expression*				
RNA Polymerase II Transcription	0.050	19/101		
Capping complex formation	0.039	7/26		
Nuclear Receptor Transcription			0.005	28/51
*Steroid hormones*				
Vitamin D (calciferol) metabolism	0.048	3/7		
*Activated AMPK stimulates fatty-acid oxidation in muscle*			0.008	12/18
*Neuronal System*				
*Heterodimerization of CEACAMs*			0.047	3/3
Transmission across Chemical Synapses			<0.001	66/108
*Immune System*				
Interleukin-2 signaling	0.029	10/41		
14-3-3 zeta binding allows recruitment of PI3K	0.033	5/15		
Signaling by interleukins			0.002	54/105
*Hemostasis*			<0.001	206/426
Platelet homeostasis	0.008	14/56		
Platelet activation, signaling and aggregation	0.011	35/187		

P-value: the p-value of the hypergeometric test unadjusted for multiple comparisons, Fraction: number of proteins in the pathway that are targets of differentially expressed miRNAs over the total number of proteins in each pathway.

## Discussion

In this paper we report the changes of urinary miRNA spectrum in T1D patients with different stages of albuminuria and nephropathy. We found concentration changes on specific miRNAs that may involve in specific pathways known to be altered in various forms of renal diseases. Since the kidney is the most likely source of these urinary miRNAs, we suggest that these miRNAs may be of biological and clinical significance in T1D.

A global Principal Component Analysis viewpoint of the microRNA profiles analyzed in this report suggests that there are some differences in the expression of urinary microRNA which appear to follow the clinical classification of patients and urinary samples with respect to albumin excretion. The apparent clustering of profiles from patients who had been matched into pairs, suggests that there are other factors affecting urinary microRNA besides the clinical classification of disease. Such factors are likely related to the variables we used in patient matching e.g. age, sex, and duration of disease and level of glycemic control. This observation justifies post-hoc our decision to explore specific microRNA signatures across the spectrum of clinical classification of patients and samples using a matched case control design.

Our matched case-control Bayesian analyses highlight a set of 27 differentially regulated miRNAs across different clinical stages of diabetic renal disease. Previous work using experimental, clinical chemistry or biopsy samples has demonstrated differential expression of many of these miRNAs in a variety of renal conditions: *hypertensive nephrosclerosis* (with an increase of miR-429 levels in human renal biopsies [25]), mouse models of chronic renal injury (increased miR-214 levels [26]) and *renal senescence* [27] (increased miR-335 levels). Other miRNAs have also been implicated in immunologically mediated renal diseases such as *lupus nephritis*(miR-429 [28], miR-638,miR-373-5p and miR-92b-5p [29]), *IgA nephropathy* (miR-429 correlating with the level of proteinuria and renal function [30]), and *acute T cell rejection of renal allografts* (decreased miR-323-5p, miR-638/miR-373-5p [31]).

Based on miRNA target prediction databases, miRNAs showing concentration changes in diabetic urine may regulate genes that play key roles in renal physiology and pathophysiology: *fibronectin* a key component of the extracellular matrix that accumulates in diabetic nephropathy [32] (miR-17-5p [33] which is also regulated in senescence models of renal proximal tubule epithelial cells [34]), *PKD2* responsible for polycystic kidney disease (miR-17-5p [35], [36]), *Sod2*, superoxide dismutase, a mitochondrial antioxidant enzyme in renal mesangial cells(miR-335 [27]), *Claudin-16* a key component of the tight junction in the thick ascending limb (has-miR-323b-5p [37]), the tumor suppressor protein PTEN which is decreased in DN [38] (and is directly regulated by miR-221-3p/222-3p [39] in heterologous systems), *Abcg2* (a stem cell marker [40] regulated by miR-520h [41]), *Vhl* (a tumor suppressor gene involved in renal tumours targeted by miR-92a [42]). Hence, prior research highlights a kidney related role for a number of the miRNAs found to be differentially expressed in our analyses, suggesting that these miRNAs may be important mediators of renal damage rather than simple biomarkers of an underlying injury process without pathobiological significance.

In addition, intriguing connections in heterologous systems have been reported for other miRNAs highlighted in this report: miR-221-3p/222-3p (neovascularization and vascular neointimal hyperplasia [43], Advanced Glycosylation End product mediated vascular damage [44]), miR-424 (regulating angiogenesis in the setting of hypoxia by targeting *Cul2* [45] as well as *Vegfr2* and *Fgfr1* [46]). Many of these conditions have been recognized as clinically important vascular complications of diabetes, often presenting simultaneously with the development of nephropathy; hence one may conjecture that the spectrum of urine miRNAs may allow one to stratify the risk of diabetic patients for developing extrarenal complications.

With the samples used in this study, we could not verify the association of miR-192 with DN. Higher miR-192 levels have been previously linked to renal damage in the streptozocin (T1D) and the db/db (T2D) mouse nephropathy models [47] through TGFβ –mediated production of miR-192 by mesangial cells. More recent evidence points towards a positive feedback loop for TGFβ production involving miR-192 and miR-200b/c in mesangial cells [48]. On the other hand, *decreased* miR-192 was noted in biopsy specimens of patients with advanced diabetic nephropathy, while miR-192 expression was positively correlated with EGFR and negatively correlated with the degree of fibrosis suggesting a protective role for miR-192 [49]. In that report, miR-192 expression was predominantly localized to tubular epithelial cells and TGF exposure was found to decrease both miR-192 and E-cadherin mRNA levels. Hence it appears that miR-192 may be regulated differently in different renal cell populations, possibly in a DN stage specific manner. This hypothesis is supported by recent evidence which failed to detect alterations in miR-192 expression in microdissected glomeruli of Munich Wistar Fromter rat model of spontaneous develop diabetic nephropathy [50]. Since the miRNAs in the urine originate from diverse cellular sources in the kidney, the lack of a differential expression of miR-192 in this report may reflect the cancelation of two diverging (positive in mesangial, negative in tubular epithelial cells) signals leading to an overall “null” effect.

Most of the identified miRNAs exhibited changes in one disease state rather than showing a quantitative trend of increasing or decreasing expression paralleling the severity of albuminuria. To understand this pattern we examined the predicted targets of these miRNAs and the corresponding pathways using structured vocabularies for biological annotation. Despite the disparate identity of the miRNAs, the mRNAs that are predicted to be targeted by them map to pathways that have been previously shown to be pathophysiologically relevant to DN: TGF (the prototypical “renal-fibrosis” culprit [51]), PDGF (associated with mesangial proliferation and fibrosis [52]) and FGF (clinical predictor of progression in diabetic nephropathy [53]).

Our analyses suggest the involvement of NGF (Nerve Growth Factor, a prototypical Central Nervous System trophic molecule) in diabetic nephropathy. This may lead to a new direction toward the development of T1D associated nephropathy since so far the renal expression of NGF has been thought to reflect the level of glycemic control [54]. Nevertheless, NGF has been recently shown to be involved in tissue repair and fibrosis in liver, skin and lung [55], and its involvement in non-diabetic renal disease has been noted in a number of biopsy studies over the last 30 years [56], so that the association of NGF with diabetic nephropathy appears plausible.

Growth Factor as well as other pathways (e.g. cell-cell and cell-matrix) are targeted from the microalbuminuric stage, while the number of targeted genes in these pathways increased at the overt nephropathy stage. Hence an “exposure-response” relation appears at the target (mRNA) rather than the regulator (miRNA) level. This relation stems from the overlapping, combinatorial, binding specificities of miRNAs to their mRNA targets so that the same pathways may be targeted by rather different sets of miRNAs depending on the prevailing cellular context.

An interesting aspect of the targets associated with the miRNAs identified in this study is the lack of an overwhelming association between growth factor transduction pathways and the *tempo* of MA. Rather, an association with tissue damage, innate immunity, metabolic pathway and developmental program (re)-activation was shown, suggesting that recurrent bouts of metabolic or free oxidative stress may account for the persistency and possibly the progression of MA to overt nephropathy. To the extent that these statistically determined patterns are verified experimentally, further development of miRNA target identification may have potential clinical implications as an early diagnostic test for diabetic renal disease or to select and or monitor response to emerging therapies for diabetic renal disease; e.g. pentoxifylline [57], pirfenidone [58] and bardoxolone [59] which interfere with pathways implicated in our analyses.

The findings of our study should be interpreted in light of a number of limitations. First, we analyzed urine samples from an era in which current therapies for diabetic nephropathy (angiotensin converting enzyme inhibitors and angiotensin receptor blockers) were not widely used early in the disease process. Hence most of the patients with MA were not on ACEi/ARB inhibition even though evidence from randomized trials suggest that these agents delay the appearance of microalbuminuria [60], [61]. On the other hand, most patients with overt nephropathy were on such agents with persistence of their macroalbuminuric state. Hence, our findings reflect the natural urinary miRNA phenotype of the early stages of diabetic nephropathy, the failing treatment one in advance disease and are not proposed to be representative of patients undergoing optimal treatment with these agents. Although this would appear to represent a major limitation of this study, the data presented here are rather unique in that they provide information on both untreated patients as well as those failing therapy, allowing some insight into the pathways that underline treatment resistance to the current treatment paradigm. This is exemplified by miR-324-3p which was apparently increased in patients with PMA not receiving an ACEi in accordance with recent animal data suggesting that this miRNA is a promoter of renal fibrosis and is downregulated by ACEi inhibition. At the same time, our patients with overt nephropathy showed no tendency of this miRNA to change relative to controls (FC was 1.06 in this dataset) suggesting that some of the discordance in miRNA profiles may be the result of therapies preferentially affecting certain miRNA species but not others. Since this investigation never intended to delineate treatment induced changes in urine miRNA profiles, future studies should examine both responders and non-responders at different points in time to determine miRNA correlates of therapeutic success and failure. Second, while our experience is no different from previous studies examining urine miRNA profiles in renal transplantation [31], [62], systemic lupus [28] and chronic kidney disease [63], many of the urinary miRNA signals in this analysis were of low magnitude requiring a large number of PCR cycles and careful optimization of qPCR conditions [64] to be detected. Third, we inferred the renal origin of urine miRNAs yet the possibility that the latter derive from other sources such as plasma cannot be ruled out. As the approximate molecular weight of miRNAs (∼6.2–7.2 kDa) is below the permselectivity threshold of the glomerular filtration barrier (∼ 60 kDa) it is possible that a substantial portion of circulating plasma miRNAs is ultrafiltered in the urine. Nevertheless, a recent study in chronic kidney disease found a dissociation between plasma and urine miRNA spectrum [63] suggesting a substantial non-plasma source for urine miRNA. To resolve these issues, simultaneous profiling of plasma and urine should be undertaken, a task which was not possible in this report due to the unavailability of plasma samples. Fourth, some of the miRNAs identified as differentially regulated have been found to play a role in non-diabetic renal disease, so that the reported associations may lack disease specificity. We tried to overcome this limitation by combining the changes in miRNA concentrations with the simultaneous predictions of miRNA targets. Most of the pathways identified have been linked to the development of diabetic nephropathy among different animal models and clinical studies which suggests the combination of using specific miRNA levels and its interacting mRNA targets as a general approach to enhance interpretability and specificity of miRNA profiles. Furthermore, the use of panels of markers will be much more informative and can potentially distinguish pathologies that produce overlapping sets of markers.

In summary, a set of 27 differentially miRNAs were identified in matched urine samples from T1D patients with different stages of diabetic nephropathy, whose renal outcomes had been ascertained after prolonged follow up. These miRNAs map to pathways of known relevance to the development of diabetic renal disease, strongly suggesting the renal source of the miRNAs. Our results suggest that a number of miRNAs in urine may serve not only as molecular signatures of distinct clinical phenotypes in diabetic nephropathy but also as early indicators of alterations in specific biological processes in the kidney which can be of importance in individualizing emergent therapies for diabetic kidney disease. Further studies are needed to extend these observations in the setting of T2D and clarify the potential utility of these miRNAs in early diagnosis, risk stratification for progression and treatment selection or monitoring.

## Supporting Information

Figure S1 **Detection probability (% of PCR reactions which yielded a signal up to a maximum of 38 cycles/all PCR reactions utilizing the same primer set) of microRNA controls classified according to patient clinical status.** hsa-miR-103/191/423-5p: endogenous microRNA controls in the Exiqon platform per manufacturer, U6/SNORD38B/SNORD49A: small RNA (non- microRNA) endogenous controls, BLANK: Empty PCR wells, UniSP6: Spiked Control (included in 50% of plates), UniSP3: Spiked Inter-plate Calibrator (included in 100% of plates).
(TIF)

Figure S2 **Raw signals (C_t_) of microRNA controls classified according to patient clinical status and plate (A or B) for each of the 53 qPCR panels used in this study.** hsa-miR-103/191/423-5p: endogenous microRNA controls in the Exiqon platform per manufacturer, U6/SNORD38B/SNORD49A: small RNA endogenous controls, BLANK: Empty PCR wells, UniSP6: Spiked Control (included in 50% of plates), UniSP3: Spiked Inter-Plate Calibrator (included in 100% of plates). Signal reproducibility appeared to be higher for the spiked-in controls than the endogenous ones; furthermore there did not appear to be a substantial inter-plate difference to justify the use of Inter-Plate Calibration.
(PNG)

Table S1 **Names and accession numbers of microRNA species analyzed in this study.** miRBase names prior to the 18^th^ release are included as well to facilitate comparison with earlier literature. Retired entries in the 18^th^ release of miRBase are marked as“DEAD”; in the case of microRNA species not present in a particular database a “NA” entry was included in the table.
(XLS)

Text S1 **Supplementary Methods and Bayesian Software Code.**
(PDF)

Dataset S1 **Un-normalized C_q_ values from individual patient experiments.**
(ZIP)

Dataset S2 **GO term enrichment analysis of targets of differentially expressed microRNAs in baseline (normoalbuminuric) urine samples from patients with PMA versus patients with IMA.**
(ZIP)

Dataset S3 **GO term enrichment analysis of targets of differentially expressed microRNAs in follow up (microalbuminuric) urine samples from patients with PMA versus patients with IMA.**
(ZIP)

Dataset S4 **GO term enrichment analysis in follow-up versus baseline samples from PMA patients.**
(ZIP)

Dataset S5 **GO term enrichment analysis of targets of differentially expressed microRNAs in urine samples from patients with overt nephropathy versus patients without nephropathy.**
(ZIP)
