# Correction: Urinary MicroRNA Profiling in the Nephropathy of Type 1 Diabetes

**DOI:** 10.1371/annotation/37e647d5-1781-4edf-86a8-e3b533c32ad9

**Published:** 2013-08-08

**Authors:** Christos Argyropoulos, Kai Wang, Sara McClarty, David Huang, Jose Bernardo, Demetrius Ellis, Trevor Orchard, David Galas, John Johnson

A second grant from the NIH was incorrectly omitted from the Funding statement. The grant number is DK047874.

