# Supplementary figures and images for: Urinary MicroRNA Profiling in the Nephropathy of Type 1 Diabetes
Source: PLoS One. 2013 Jan 24;8(1):e54662. doi: 10.1371/journal.pone.0054662 (PMC3554645; doi:10.1371/journal.pone.0054662)

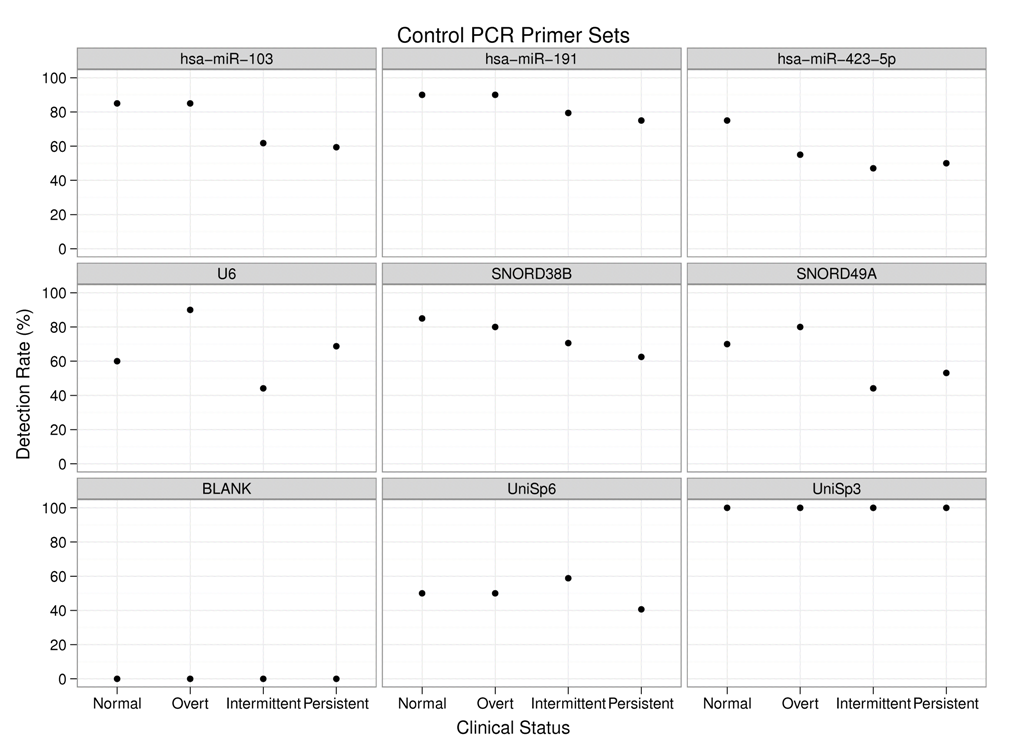

Supplement: Figure S1 — Detection probability (% of PCR reactions which yielded a signal up to a maximum of 38 cycles/all PCR reactions utilizing the same primer set) of microRNA controls classified according to patient clinical status. hsa-miR-103/191/423-5p: endogenous microRNA controls in the Exiqon platform per manufacturer, U6/SNORD38B/SNORD49A: small RNA (non- microRNA) endogenous controls, BLANK: Empty PCR wells, UniSP6: Spiked Control (included in 50% of plates), UniSP3: Spiked Inter-plate Calibrator (included in 100% of plates). (TIF) [file pone.0054662.s001.tif]

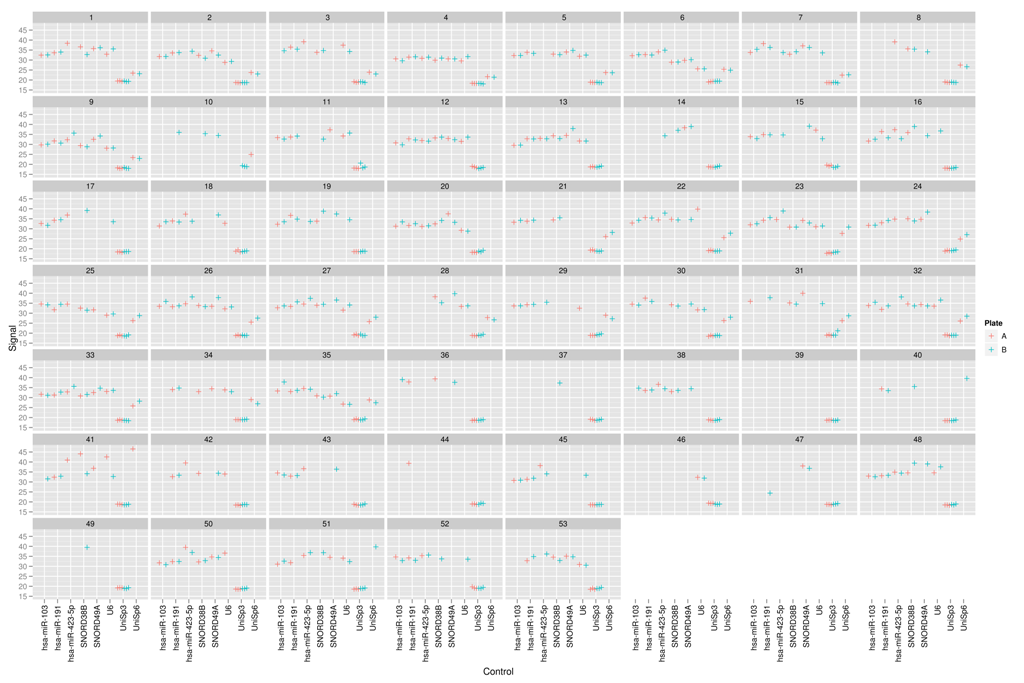

Supplement: Figure S2 — Raw signals (Ct) of microRNA controls classified according to patient clinical status and plate (A or B) for each of the 53 qPCR panels used in this study. hsa-miR-103/191/423-5p: endogenous microRNA controls in the Exiqon platform per manufacturer, U6/SNORD38B/SNORD49A: small RNA endogenous controls, BLANK: Empty PCR wells, UniSP6: Spiked Control (included in 50% of plates), UniSP3: Spiked Inter-Plate Calibrator (included in 100% of plates). Signal reproducibility appeared to be higher for the spiked-in controls than the endogenous ones; furthermore there did not appear to be a substantial inter-plate difference to justify the use of Inter-Plate Calibration. (PNG) [file pone.0054662.s002.png]
