# Supplementary material for: Urinary MicroRNA Profiling in the Nephropathy of Type 1 Diabetes
Source: PLoS One. 2013 Jan 24;8(1):e54662. doi: 10.1371/journal.pone.0054662 (PMC3554645; doi:10.1371/journal.pone.0054662)
Supplement: Text S1 — Supplementary Methods and Bayesian Software Code. (PDF) [file pone.0054662.s004.pdf]

Supplementary Methods

Bayesian Computations

microRNA C_q values in the Exiqon^{TM} qPCR platform

The Exiqon^{TM} qPCR platform offers a two step procedure for the quantification of microRNAs, by combining a *universal* reverse transcript reaction (first step) with LNA^{TM}(Locked Nucleic Acid) enhanced PCR primers. In LNA^{TM} molecules a methylene bridge connects the 2'-O atom and the 4'-C atome locking the ribose ring; incorporation of LNA^{TM}monomers in nucleic acid duplexes, increases the melting temperature by 2-8 °C/LNA^{TM}monomer, thus improving the thermal stability of the complex and increasing the binding affinity and the hybridization specificity for short target sequences such as microRNAs. Due to the universal amplification step one would expect all that signals from RNAs analyzed in the same panel to be related, calling for a normalization procedure to address this co-variability. This normalization was effected simultaneously with the estimation of the ΔC_qvalues using linear mixed effects (LME) regression models. These models were specified separately for each of the three comparisons considered in the manuscript in the WinBUGS programming language and estimated using Monte Carlo Markov Chain simulations.

Matched samples from patients with microalbuminuria (MA)

This analysis concerns microRNA profiles in patients who will develop either intermittent (IMA) or persistent (PMA) microalbuminuria at two different occasions: a) before the development of MA i.e. when the urine appears to be "normal" by conventional clinical laboratory testing and b) upon development of microalbuminuria. For this analysis, a hierarchical linear mixed model was used accounting for the explicit matching between MA patients in pairs, as well as the implicit matching between normoalbuminuric and microalbuminuric samples from the same patient was used. By employing a random effect for patient pairs, the latter were allowed to have their own specific expression for each microRNA of interest, constrained to be normally distributed around the overall mean (random intercept model). Such a modification allows the model to account for confounding factors co-varying with the variables used in matching (age, sex, diabetic control i.e. level of Hemoglobin A_1C) that could potentially affect microRNA expression. The fixed effects part of the regression equations in this model reflect the clinical classification of the patients (IMA v.s. PMA) and the laboratory classification of the corresponding samples (normoalbuminuric or microalbuminuric) through indicator variables:

The corresponding set of regression coefficients is shown in Table 2:

Tab. 1: Indicator variables used in the analysis of samples from patients with microalbuminuria

Patient Group	Sample Classification	X_i^{bP}	X_i^{fI}	X_i^{fP}
IMA	Normoalbuminuric	0	0	0
PMA	Normoalbuminuric	1	0	0
IMA	Microalbuminuric	0	1	0
PMA	Microalbuminuric	1	0	1

Tab. 2: Regression coefficients in the analysis of samples from patients with microalbuminuria

Regression Coefficient	Interpretation
$\beta_k^{c,bP}$	ΔC_q between PMA and IMA in normoalbuminuric (baseline) urine samples
$\beta_k^{c,fI}$	ΔC_qbetween microalbuminuric (follow-up) and normoalbuminuric (baseline) urine samples in IMA patients
$\beta_k^{c,fP}$	ΔC_qbetween microalbuminuric (follow-up) and normoalbuminuric (baseline) urine samples in PMA patients

By redefining the contrast of the design matrix implied by the indicator variables in Table 1 one can obtain a the new set of regression coefficients with slightly different interpretation (shown along with their relation to the coefficients of Table 2):

Tab. 3: Equivalent regression analysis of samples from IMA and PMA patients

Indicator Variable	Regression Coefficient	Interpretation
$Z_i^{bP} = X_i^{bP}$	$\beta_k^{c,bP}$	ΔC_q between PMA and IMA in normoalbuminuric (baseline) urine samples
$Z_i^{MA} = X_i^{fI}$	$\beta_k^{c,MA} = \beta_k^{c,fI}$	ΔC_qbetween microalbuminuric (follow-up) and normoalbuminuric (baseline) urine samples in IMA patients
$Z_i^{MA\times P} = X_i^{fI} + X_i^{fP}$	$\beta_k^{c,MA\times P} = \beta_k^{c,fP} - \beta_k^{c,fI}$	ΔC_qbetween microalbuminuric urine samples in PMA patients relative to microalbuminuric urine samples in the IMA group

When fitting the model we adopted the parameterization of Table 1 due to the higher numerical stability and better convergence of the Monte Carlo simulations. In order to compare microalbuminuric samples between patients with PMA and IMA without refitting, we formed the difference between the the second and the third coefficients of Table 2 after convergence had been achieved.

The adopted model assumes the quantification cycle value of each of the control reactions (Y_i^c) to be normally distributed around its mean μ_i^c with a standard deviation σ_w(measurement noise):

$$Y_i^c \sim N(\mu_i^c, \sigma_w^2)$$

The mean is related to an experimental panel factor, $E^c(ID_i)$, and a control PCR specific factor $B^c(ID_i, R_i)$ according to the regression equation:

$$\mu_i^c = E^c(ID_i) + B^c(ID_i, R_i)$$

where ID_i is the individual sample the ith control measurement came from, while R_i indexes the well number of the three microRNAs (*hsa-miR-423-5p, hsa-miR-103, hsa-miR-191*) the three small RNAs (*U6, SNORD38B,*

SNORD39A)[1] and the interplate calibrator *UniSP3*. The panel factors were modelled as *normal* random effects:

$$E^c(j) \sim N(0, \sigma_B^2), \quad j = 1, \ldots, N^c$$

which were used to effect a panel specific normalization of non-control C_q values. In this equation, the standard deviation σ_B can be interpreted as the magnitude of the noise in quantification cycle values due to the universal amplification step and run-to-run variability in signal acquisition by the real time PCR system. The control PCR specific factors are modelled as an additional level in the hierarchical model by exploiting the replicate PCR reactions (N_R reactions in each plate using N_P distinct primer sets). In the ith individual sample these are assumed to be normally distributed around their mean $\mu^{PCR}_{i,R_{miR}(j)}$ with a standard deviation $\sigma^{ID}_{RmiR(j)}$:

$$B^c(i,j) \sim N(\mu^{PCR}_{i,R_{miR}(j)}, \sigma^{ID}_{R_{miR}(j)}), \quad i = 1, \ldots, N_{ID}, \quad j = 1, \ldots, N_R$$

in which $R_{miR(j)}$ maps the jth PCR reaction to the corresponding primer set. To account for clinical, and laboratory classification of samples and the matching between pairs, the means were resolved with the aid of the regression:

$$\begin{aligned} \mu^{PCR}_{i,k} &= \alpha^c_k +_a P^c_{Pair_i,k} + \beta^{c,bP}_k \cdot X^{bP}_i + \beta^{c,fI}_k \cdot X^{fI}_i + \beta^{c,fP}_k \cdot X^{fP}_i, \quad k = 1, \ldots, N_P \\ {}_aP^c_{j,k} &\sim N(0, \sigma^c_i), \quad k = 1, \ldots, N_P, \quad j = 1, \ldots, N_{pairs} \end{aligned}$$

in which α^c_k is the expression level of the kth control reaction and ${}_aP^c_{j,k}$ is the (random) intercept of the kth control reaction in the the jth pair of patients. A slightly different regression was used for the analysis of the non-control PCR reactions since these were assayed only once in each sample. The N non-control measurements (from all patient samples) map to N_{miR} unique microRNAs which are assumed to be normally distributed around their mean with the same standard deviation (measurement noise) as the control reactions:

$$\begin{aligned} \mu_i &= \beta_{Pair_i,miR(i)} + \beta^{bP}_k \cdot X^{bP}_{miR(i)} + \beta^{fI}_k \cdot X^{fI}_{miR(i)} + \beta^{fP}_k \cdot X^{fP}_{miR(i)} + E^c(ID_i), \quad i = 1, \ldots, N \\ \beta_{j,k} &\sim N(\alpha_k, \sigma_k), \quad k = 1, \ldots, N_{miR}, \quad j = 1, \ldots, N_{pairs} \end{aligned}$$

Similar to the control PCR reactions, indicator variables are used to classify the corresponding urine sample according to clinical and laboratory classification. For the non-control reactions, the panel specific factors appear as *offsets* that adjust individual measurements for the variable efficiency in the universal amplification step. Since these factors are not observed, they are treated as *missing data* which are estimated from the control reactions. This corresponds to a sequential two stage model for missing data but with full allowance for uncertainty in the estimation of unknown quantities.

In this analysis, non-informative priors were specified for the model parameters (regression coefficients, standard deviations of random effects):

$$\begin{aligned} \alpha^c_k &\sim N(0, 10^6) & \alpha_k &\sim N(0, 10^6) \\ \beta^{c,bP}_k &\sim N(0, 10^6) & \beta^{bP}_k &\sim N(0, 10^6) \\ \beta^{c,fI}_k &\sim N(0, 10^6) & \beta^{fI}_k &\sim N(0, 10^6) \\ \beta^{c,fP}_k &\sim N(0, 10^6) & \beta^{fP}_k &\sim N(0, 10^6) \\ \sigma^{ID}_k &\sim U(0, 100) & \sigma_k &\sim U(0, 100) \\ \sigma^c_k &\sim U(0, 100) \end{aligned}$$

$$\begin{aligned} \sigma_B &\sim U(0, 100) \\ \sigma_w &\sim U(0, 20) \end{aligned}$$

The values of the standard deviation of the normal priors for the mean parameters (1000) and the upper limit of the uniform priors for standard deviations (100 and 20) were motivated by numerical considerations as they yield essentially non-informative priors in the context of the finite precision arithmetic of digital computations.

[1] Although suggested as reference genes (biological controls) by the panel manufacturer the 6 microRNAs/small nuclear RNAs were not used as such in this analysis. Hence their designation as controls is only used as a means to differentiate the replicated PCR reactions from the rest of the microRNAs which were only assayed once in each panel.

Matched samples from patients with overt nephropathy and normals

This analysis is similar to the one carried out for patients with microalbuminuria in that a random effects are used to account for the matching of patients in pairs. The only difference lies in the fixed effects part of the model, i.e. the indicator variable ($X_i^{Ov} = 1$ if the sample came from a patient with overt nephropathy and 0 otherwise) and the corresponding regression coefficient (interpretable as ΔC_qqPCR signal for overt nephropathy relative to the normal state):

$$\begin{aligned} \mu_{i,k}^{PCR} &= \alpha_k^c +_a P_{Pair_i,k}^c + \beta_k^{c,Ov} \cdot X_i^{Ov}, \quad k = 1, \ldots, N_P \\ \mu_i &= \beta_{Pair_i,miR(i)} + \beta_k^{Ov} \cdot X_{miR(i)}^{Ov} + E^c(ID_i), \quad i = 1, \ldots, N \end{aligned}$$

Non-informative $N(0, 10^6)$ priors were specifed for the nephropathy effect for both control and non-control PCRs, as well as for the remaining model parameters similar to the previous sections.

Calculation of $\Delta\Delta C_q$ values

Each of the three models allow the estimation of ΔC_qvalues for all RNAs of interest assayed in the experiment. To calculate the $\Delta\Delta C_q$values, one forms the difference between the ΔC_q for the kth microRNA (given by a "beta" coefficient in the sample comparison contemplated) and the $\Delta C_q$for UniSP3. Since both ΔC_q values are estimated from the data, these differences are averaged over the posterior distribution of these quantities given the raw C_q signals. The corresponding integral can be approximated by forming the differences between the corresponding ΔC_q samples from the Markov Chain Monte Carlo simulations. Means and Standard Errors of these $\Delta\Delta C_q$s were used in the meta-analysis of microRNA targets.

WinBUGS Code

WinBUGS code for the Bayesian estimation of the C_q in three analyses are given in the listings below. In WinBUGS the normal distribution is parameterized in terms of the precision (inverse of the square of the standard deviation), necessitating the introduction of auxilliary variables in the code. The *cut* function was used in the code to ensure that only data from the control PCR reactions were used to "learn" the values of the panel specific normalization factors. During Monte Carlo Markov Chain (MCMC) simulation for the estimation of the *quantification cycle* (threshold crosing) values of the non-control PCR reactions, this uncertainty was taken fully into account by marginalizing over the posterior distribution of $E^c(ID_i)$ obtained by conditioning on the control data. For all models, we simulated three chains with overdispersed initial values and explored convergence both by visual inspection and formal convergence diagnostics (Gelman-Rubin). The first 10000 iterations of the simulations were discarded (*burn-in* phase) and subsequently 200000 draws were obtained in WinBUGS. Only one draw in 100 was stored and used further, eliminating for all practical purposes the autocorrelation between consecutive samples. WinBUGS MCMC output was then imported in the *R* programming language for further processing (calculation of means and standard errors of $\Delta\Delta C_q$s, Credible Interval and pseudocontour probabilities computations, meta-analysis etc.).

Analysis of Samples from Patients with MicroAlbuminuria

```
model {
##=============================================================================
##      Part of the model relating to control reaction and miRs - LME
##=============================================================================

        for(i in 1:cntr.N)      {
                cntr.Y[i] ~ dnorm(cntr.mu[i],tau.within)
                cntr.mu[i] <- cntr.exp[cntr.ID[i]] + cntr.b[cntr.ID[i],cntr.R[i]]
        }

        for(i in 1:NID) {
        ## iterate over the number of control reactions (NR)
        ## R2miR maps reaction to control miR
                for(j in 1:NR)  {
                        cntr.b[i,j] ~ dnorm(cntr.PCR[i,j],tauID[R2miR[j]])
                        cntr.PCR[i,j] <- cntr.alpha[R2miR[j]] +
                                         cntr.alphaP[cntr.Pair[i],R2miR[j]] +
                                         cntr.XStage[i,1]*cntr.bu[R2miR[j],1] +
                                         cntr.XStage[i,2]*cntr.bu[R2miR[j],2] +
                                         cntr.XStage[i,3]*cntr.bu[R2miR[j],3]
                }
                ## tauB interpretable as noise due to amplification factors
                cntr.exp[i] ~ dnorm(0.0,tauB)
                cut.exp[i] <- cut(cntr.exp[i])
        }

        for(i in 1:NPair)       {
                for(j in 1:cntr.NmiR)   {
                        cntr.alphaP[i,j] ~ dnorm(0.0,cntr.tau[j])
                }
        }

        for(i in 1:cntr.NmiR)   {
                cntr.alpha[i] <- alpha[NmiR+i]
                cntr.bu[i,1]  <- cntr.bSP[i]
                cntr.bu[i,2]  <- cntr.fuI[i]
                cntr.bu[i,3]  <- cntr.fuP[i]

                cntr.bSP[i] <- BsP[NmiR+i]
                cntr.fuI[i] <- FuI[NmiR+i]
                cntr.fuP[i] <- FuP[NmiR+i]

                sigmaID[i] <- 1/sqrt(tauID[i])

                tauID[i] <- pow(sigID[i],-2)
                sigID[i] ~ dunif(0.0,100.0)

                cntr.tau[i] <- pow(cntr.sigma[i],-2)
                cntr.sigma[i] ~ dunif(0.0,100.0)
        }

        tauB <- pow(sigmaB,-2)
```

```
        sigmaB ~ dunif(0.0,100.0)

##==========================================================================
##      Part of the model relating to non control reaction and miRs
##==========================================================================

        for(i in 1:N)   {
                Y[i] ~ dnorm(mu[i],tau.within)
                mu[i] <- b[Pair[i],miR[i]]+XStage[ID[i],1]*bAlbu[miR[i],1] +
                                           XStage[ID[i],2]*bAlbu[miR[i],2] +
                                           XStage[ID[i],3]*bAlbu[miR[i],3] +
                        cut.exp[ID[i]]
        }

        for(i in 1:NPair)       {
                for(j in 1:NmiR)        {
                        b[i,j]~dnorm(alpha[j],tau[j])
                }
        }
        for(i in 1:NmiR)        {
                bAlbu[i,1] <- BsP[i]
                bAlbu[i,2] <- FuI[i]
                bAlbu[i,3] <- FuP[i]

                sigma[i] ~ dunif(0.0,10)
                tau[i] <- pow(sigma[i],-2)
        }

        for(i in 1:NmiR+cntr.NmiR)      {
                alpha[i] ~ dnorm(0.0,1.0E-6)
                BsP[i] ~ dnorm(0.0,1.0E-6)
                FuI[i] ~ dnorm(0.0,1.0E-6)
                FuP[i] ~ dnorm(0.0,1.0E-6)
        }

        tau.within <- pow(sigma.within,-2)
        sigma.within ~ dunif(0,20)
}
```

Overt Nephropathy v.s. Normal

```
model {
##===========================================================================
##      Part of the model relating to control reaction and miRs - LME
##===========================================================================

        for(i in 1:cntr.N)      {
                cntr.Y[i] ~ dnorm(cntr.mu[i],tau.within)
                cntr.mu[i] <- cntr.exp[cntr.ID[i]] + cntr.b[cntr.ID[i],cntr.R[i]]
        }

        for(i in 1:NID) {
        ## iterate over the number of control reactions (NR)
        ## R2miR maps reaction to control miR
                for(j in 1:NR)  {
                        cntr.b[i,j] ~ dnorm(cntr.PCR[i,j],tauID[R2miR[j]])
                        cntr.PCR[i,j] <- cntr.alpha[R2miR[j]] +
                                         cntr.alphaP[cntr.Pair[i],R2miR[j]] +
                                         cntr.XStage[i]*cntr.bu[R2miR[j],1]
                }
                ## tauB interpretable as noise due to amplification factors
                cntr.exp[i] ~ dnorm(0.0,tauB)
                cut.exp[i] <- cut(cntr.exp[i])
        }

        for(i in 1:NPair)       {
                for(j in 1:cntr.NmiR)   {
                        cntr.alphaP[i,j] ~ dnorm(0.0,cntr.tau[j])
                }
        }

        for(i in 1:cntr.NmiR)   {
                cntr.alpha[i] <- alpha[NmiR+i]
                cntr.bu[i,1]    <- cntr.bS3[i]

                cntr.bS3[i] <- Bt3[NmiR+i]

                sigmaID[i] <- 1/sqrt(tauID[i])

                tauID[i] <- pow(sigID[i],-2)
                sigID[i] ~ dunif(0.0,100.0)

                cntr.tau[i] <- pow(cntr.sigma[i],-2)
                cntr.sigma[i] ~ dunif(0.0,100.0)
        }

        tauB <- pow(sigmaB,-2)
        sigmaB ~ dunif(0.0,100.0)

##===========================================================================
##      Part of the model relating to non control reaction and miRs
##===========================================================================
```

```
        for(i in 1:N)    {
                Y[i] ~ dnorm(mu[i],tau.within)
                mu[i] <- b[Pair[i],miR[i]]+XStage[ID[i]]*bAlbu[miR[i],1] +
                        cut.exp[ID[i]]
        }

        for(i in 1:NPair)        {
                for(j in 1:NmiR)         {
                        b[i,j]~dnorm(alpha[j],tau[j])
                }
        }
        for(i in 1:NmiR)         {
                bAlbu[i,1] <- Bt3[i]

                sigma[i] ~ dunif(0.0,10)
                tau[i] <- pow(sigma[i],-2)
        }

        for(i in 1:NmiR+cntr.NmiR)       {
                alpha[i] ~ dnorm(0.0,1.0E-6)
                Bt3[i] ~ dnorm(0.0,1.0E-6)
        }
}

        tau.within <- pow(sigma.within,-2)
        sigma.within ~ dunif(0,20)
}
```
